# Compound dark tea ameliorates obesity and hepatic steatosis and modulates the gut microbiota in mice

**DOI:** 10.3389/fnut.2023.1082250

**Published:** 2023-01-20

**Authors:** Jianyu Qu, Mengke Ye, Chi Wen, Xianyu Cheng, Lirui Zou, Mengyao Li, Xiangyan Liu, Zhonghua Liu, Lixin Wen, Ji Wang

**Affiliations:** ^1^Hunan Engineering Research Center of Livestock and Poultry Health Care, Colleges of Veterinary Medicine, Hunan Agricultural University, Changsha, China; ^2^Hunan Chu Ming Tea Industry Co., Ltd., Changsha, China; ^3^Key Laboratory of Tea Science of Ministry of Education, National Research Center of Engineering Technology for Utilization of Functional Ingredients from Botanicals, College of Horticulture, Hunan Agricultural University, Changsha, China; ^4^Animal Nutritional Genome and Germplasm Innovation Research Center, College of Animal Science and Technology, Hunan Agricultural University, Changsha, China; ^5^Changsha Lvye Biotechnology Co., Ltd., Changsha, China

**Keywords:** compound dark tea, gut microbiota, obesity, lipid metabolism, hepatic steatosis

## Abstract

Dark tea is a fermented tea that plays a role in regulating the homeostasis of intestinal microorganisms. Previous studies have found that dark tea can improve obesity and has a lipid-lowering effect. In this study, green tea, *Ilex latifolia Thunb* (kuding tea) and *Momordica grosvenori* (Luo Han Guo) were added to a new compound dark tea (CDT), to improve the taste and health of this beverage. High-fat diet-fed C57BL/6J mice were treated with low- (6 mg/mL) or high- (12 mg/mL) concentrations of CDT for 18 weeks to assess their effect on lipid metabolism. Our results suggest that low- and high-concentrations of CDT could reduce body weight by 15 and 16% and by 44 and 38% of body fat, respectively, by attenuating body weight gain and fat accumulation, improving glucose tolerance, alleviating metabolic endotoxemia, and regulating the mRNA expression levels of lipid metabolism-related genes. In addition, low concentrations of CDT were able to reduce the abundance of *Desulfovibrio*, which is positively associated with obesity, and increase the abundance of *Ruminococcus*, which are negatively associated with obesity. This study demonstrates the effect of CDT on ameliorating lipid metabolism and provides new insights into the research and development of functional tea beverages.

## 1. Introduction

Obesity, which causes glucose and lipid metabolism disorder by excessive accumulation of adipose tissue in the body, further leads to the occurrence of chronic metabolic diseases such as atherosclerosis, diabetes, and non-alcoholic fatty liver (1). Currently, there are approximately one billion people with obesity worldwide (2). A 25% prevalence of non-alcoholic fatty liver disease (NAFLD) has been reported worldwide (3). The estimated prevalence of NAFLD in children and adolescents with obesity is 36.1% (4). The prevalence of NAFLD in children and adolescents is expected to increase along with the global obesity epidemic. Therefore, improving and preventing obesity is a major challenge for us. In recent years, an increasing number of studies have found that gut microbiota plays an important role in human health. Intestinal flora disorders are closely linked to diseases, such as diabetes, non-alcoholic fatty liver disease and obesity (5). In addition, intestinal flora disorders can alter the production of gastrointestinal peptides associated with satiety, leading to increased food intake (6). Studies have shown that dietary foods and their functional components can alter the structure and composition of the gut microbiota. For example, probiotics containing prebiotic components reduce body mass index (BMI) and body fat levels through the gut microbiota (7). Similarly, fecal transplantation experiments have shown that the gut microbiota of mice fed with resveratrol can reduce obesity in high-fat diet (HFD)-fed mice (8). Thus, gut microbes have been established as possible therapeutic targets for preventing or treating overweight, obesity, and diseases linked to these conditions.

Tea, one of the most popular beverages in the world, contains a variety of bioactive ingredients that is beneficial to human health, such as polysaccharides, polyphenols, and catechins (9). As a dietary structure, tea exhibits antioxidative, anti-inflammatory, antimicrobial, anticarcinogenic, antihypertensive, neuroprotective, cholesterol-lowering, lipid-lowering, blood glucose-lowering, and thermogenic properties (10, 11). According to the different treatment method, tea is mainly divided into unfermented, semifermented, and fully fermented teas. As an unfermented tea, green tea can change the serum and liver metabolomic profiles of mice with high-fat diet-induced obesity (12). It has been reported that caffeine and catechins in green tea increase thermogenesis and fatty oxidation substrates by affecting the sympathetic nervous system (13). As a fully fermented tea, *Ilex latifolia Thunb* (Kuding tea) is a traditional bitter-tasting herbal tea that has been widely recognized over the past decade for its antihypertensive, lipid-lowering, blood-glucose-lowering, and antiobesity properties by the major bioactive components of triterpene saponins and polyphenols (14). Dark tea, including Pu'er, Liupao, and Fu brick teas, is also a fully fermented tea and has been gradually favored by consumers in recent years. It has been reported that Pu'er tea exerts lipid-lowering effects. Moreover, genetic and microbiology studies have discovered that some biomarkers of diabetes and obesity have improved following Pu'er tea intervention (15). Liupao tea, a dark tea, can alleviate symptoms associated with obesity by regulating lipid metabolism and oxidative stress, including lipid metabolism disorders, liver damage, and chronic inflammation (16). Fu brick tea is fermented by the solid-state fermentation of tea leaves with the probiotic *Eurotium cristatum* (golden flora) as the only dominant fungus (17). As the main active components of Fu brick tea, polyphenols are converted to new products by *Eurotium cristatum* (golden flora) during solid-state fermentation, including A-ring and B-ring fission derivatives of flavan-3-ols such as fuzhuanins A–F, teadenol A, teadenol B, xanthocerin, and planchol A (18–21). Fu brick tea can effectively reduce obesity in HFD rats and improve intestinal flora disorders, inflammation, and oxidative stress (22). In addition, drinking dark tea can attenuate systemic metabolic endotoxemia (23). Tea has been extensively studied in recent decades for its beneficial health effects in preventing obesity, metabolic syndrome, diabetes, and other diseases (24). Although these beneficial effects have been demonstrated in most laboratory studies, they are difficult to apply in real life. For example, green tea, *Ilex latifolia Thunb* (kuding tea), and dark tea drinks can have good weight loss results (22, 25–27), but we ignore the high concentration of tea that has bitter and astringent tastes to reduce its palatability (28).

Therefore, we developed a novel functional compound dark tea, to solve this problem. To increase the palatability of functional compound dark tea (CDT), including Fu brick tea, green tea, and *Ilex latifolia Thunb* (kuding tea), we added the *Momordica grosvenori* (Luo Han Guo). As a natural sweetener, *Momordica grosvenori* (Luo Han Guo) has been used as a sugar substitute for those with obesity and those with diabetes (29). *Momordica grosvenori* (Luo Han Guo) has also been used to prepare hot drinks to treat sore throat and as an expectorant for thousands of years in China (30). Our study would improve the taste and antiobesity effects of dark tea, we developed a compound dark tea (CDT) based on Fu brick tea, adding green tea, *Ilex latifolia Thunb* (kuding tea) and *Momordica grosvenori* (Luo Han Guo) to explore the effect and mechanism of CDT on lipid metabolism and gut microbiota in mice.

## 2. Materials and methods

### 2.1. Preparation and characterization of CDT

The main ingredients of CDT include Fu brick tea (50%), green tea (25%), *Ilex latifolia Thunb* (kuding tea) (10%) and *Momordica grosvenori* (Luo Han Guo) (15%), which were purchased from Hunan Chu Ming Tea Industry Co., Ltd. (Hunan, China). The preparation of CDT aqueous extracts was as follows: low concentration CDT (6 mg/mL, CDT_L group), 1.8 g CDT was soaked in 300 mL boiling water for 25 min, then filtered out the tea residue and allowed the mixture to cool; high concentration CDT (12 mg/mL, CDT_H group), 3.6 g CDT was soaked in 300 mL boiling water for 25 min, then filtered out the tea residue and allowed the mixture to cool.

### 2.2. Diet, animals, and treatment

Mouse diet preparation was performed by Trophic Animal Feed High-tech Co., Ltd. (Jiangsu, China). Diet composition and calorie levels in mice are shown in Supplementary Tables S1, S2. In addition, 36 male C57BL/6J mice (6 weeks old) were purchased from Hunan Slake Jingda Experimental Animals Co., Ltd. (Hunan, China). All mice were maintained at a controlled temperature of 22 ± 2 °C, humidity of 65 ± 5%, and a 12-h light/dark cycle. All the mice had *ad libitum* access to food and water. After 1 week of adaptive feeding, 6-week-old male C57BL/6J mice were randomly divided into four groups: the Con (including the common diet group, *n* = 9), HFD (HFD group, *n* = 9), CDT_L (HFD + low-concentration CDT group, *n* = 9), and CDT_H (HDF + high-concentration CDT group, *n* = 9) groups. Mice in the CDT_L and CDT_H groups all received drinking CDT in sterile plastic bottles, which were replaced every 2 days with freshly made low- and high-concentration CDT, with the surplus being collected and measured. Food consumption and body weight were recorded once a week for 18 weeks. At the end of the experiment, the mice were anesthetized with tribromoethanol after 8 h of fasting. The cecum contents, liver, serum, perirenal, epididymal, subcutaneous, and brown adipose tissues were collected for follow-up experiments, immediately frozen in liquid nitrogen, and stored at −80 °C. The experimental procedures were performed following the Animal Care and Use Guidelines of China and were approved by the Animal Care Committee of Hunan Agricultural University and the Use Committee at HUNAU (No. 43322108).

### 2.3. Oral glucose tolerance test

After 18 weeks, an oral glucose tolerance test (OGTT) was performed as previously described (31). Briefly, the mice were fasted for 6 h, followed by oral administration of glucose (2.0 g/kg body weight). Then, the blood glucose concentrations were analyzed at 0, 30, 60, 90, and 120 min after the oral administration of glucose, and a glucose concentration-time plot was prepared to offer the integrated areas under each curve (AUC) for OGTT. The glucose levels were measured using an ACCU-CHEK glucose meter (Roche, Shanghai, China). The data were recorded and analyzed.

### 2.4. Biochemical assessment

The serum levels of triglyceride (TG), total cholesterol (TC), low-density lipoprotein cholesterol (LDL-C), high-density lipoprotein cholesterol (HDL-C), alkaline phosphatase (ALP), and aspartate aminotransferase (AST) were determined using a model BS-240 automatic biochemical analyzer (Shenzhen Mindray Bio-Medical Electronics, Shenzhen, Guangdong, China). Liver TG, TC, and FFA levels were assessed using commercial kits (Beijing Box Shenggong Technology Co., Ltd., Beijing, China). The serum levels of insulin, resistin, leptin, and glucagon-like peptide 1 (GLP-1) were assessed using commercial kits (Supplementary Table S3). Additionally, fasting blood glucose content was measured using an Accu-Chek Performa glucose meter (Roche, Shanghai, China). The fasting blood glucose and fasting insulin values were used to calculate the homeostasis model assessment of insulin resistance (HOMA-IR) index as follows: HOMA-IR = (fasting glucose × fasting insulin)/22.5 (32).

### 2.5. Histological analysis

Subcutaneous adipose, brown adipose, and liver samples were cut into small pieces, dehydrated, cleared, embedded in paraffin wax, and sectioned after fixation in 10% neutral-buffered paraformaldehyde. Sections (5 mm) of each sample were prepared using hematoxylin and eosin (H&E) staining methods. To validate the vacuolization analysis and quantify lipid droplets, frozen liver samples were sectioned, stained with 0.2% oil red O in 60% isopropanol, and washed three times with phosphate-buffered saline (PBS). Images of stained liver tissues were collected using an orthographic light microscope (Nikon).

### 2.6. Quantification of brown adipose with immunohistochemistry

Brown adipose tissue was fixed in 4% paraformaldehyde and embedded in paraffin. Each sample was sliced into five sections discontinuously and then used for evaluation of relative brown adipose tissue by immunohistochemistry. After antigen repair and blockage of the endogenous peroxidase, sections were washed in PBS three times for 10 min and then incubated overnight at 4 °C with a rabbit antibody specific for GLP-1 (1:200, AiFang biological, AF11181). Tissue sections were washed three times in PBS for 10 min and incubated for 2 h at room temperature with the secondary antibody (HRP-Polymer anti-mouse/rabbit universal IHC kit, AiFang biological, AFIHC001, Changsha, China). After washing in PBS three times, tissue sections were stained with the chromogenic substrate 3,3′-diaminobenzidine (DAB), and the positive cell number was observed under the microscope. The number of positive cells was recorded at every high magnification, and the average of each sample was calculated. Sections from five animals from different treatment groups were analyzed (33).

### 2.7. Quantitative real-time polymerase chain reaction

Total RNA was isolated using TRIzol reagent (Accurate Biology, Hunan, China). RNA was then converted into complementary DNA (cDNA) using the Evo M-MLV Reverse Transcription Kit (Accurate Biology). Reverse transcription-quantitative polymerase chain reaction (RT-qPCR) assays were performed using the SYBR Green Premix Pro Taq HS qPCR Kit (AG11701; Accurate Biology, Hunan, China) and on a high-throughput PCR instrument (WaferGen Biosystems, Fremont, CA, USA). Thermal cycling conditions were as follows: one cycle at 95 °C for 10 min, 95 °C for 30 s, and 60 °C for 30 s. Expression levels of genes were normalized to β-actin expression. The quantitative changes in gene expression were calculated using the 2–ΔΔCt method, where Ct = Ct (target gene) – Ct (β-actin). The primer sequences are listed in Supplementary Table S4.

### 2.8. Gut microbiota analysis

The intestinal microbiota diversity detection experiment was commissioned by Majorbio Bio-Pharm Technology Co., Ltd. (Shanghai, China) based on the Illumina MiSeq PE300 platform. The specific operation was the same as that in previous research methods (34). Bacterial genomic DNAs in feces (*n* = 7 per group) were sequenced by Majorbio Bio-Pharm Technology Co., Ltd. (Shanghai, China). Alpha diversity, including the Shannon and Sobs indexes, was calculated using Mothur (version 1.30.2). Comparative analysis of samples between groups was performed using partial least squares discriminant analysis (PLS-DA). Spearman's correlation analysis was conducted to explore the correlation between bacterial flora and physiological factors.

### 2.9. Statistical analysis

The results were presented as the mean ± standard error of the mean (SEM). Statistical Package for the Social Sciences (SPSS) (version 25.0; IBM SPSS, Chicago, IL, USA) and GraphPad Prism (version 9.0; GraphPad Software, San Diego, CA, USA) were used for the statistical analyses. Data were tested for normality using the Shapiro–Wilk test. Normally distributed data were analyzed using a one-way analysis of variance (ANOVA). Non-normally distributed data were analyzed using the Kruskal–Wallis rank-sum test. Statistical significance was set at *P* < 0.05, while *P* < 0.01 was considered highly significant.

## 3. Results

### 3.1. Effect of CDT on glucose-lipid metabolism in HFD-fed mice

**Figure 1:**
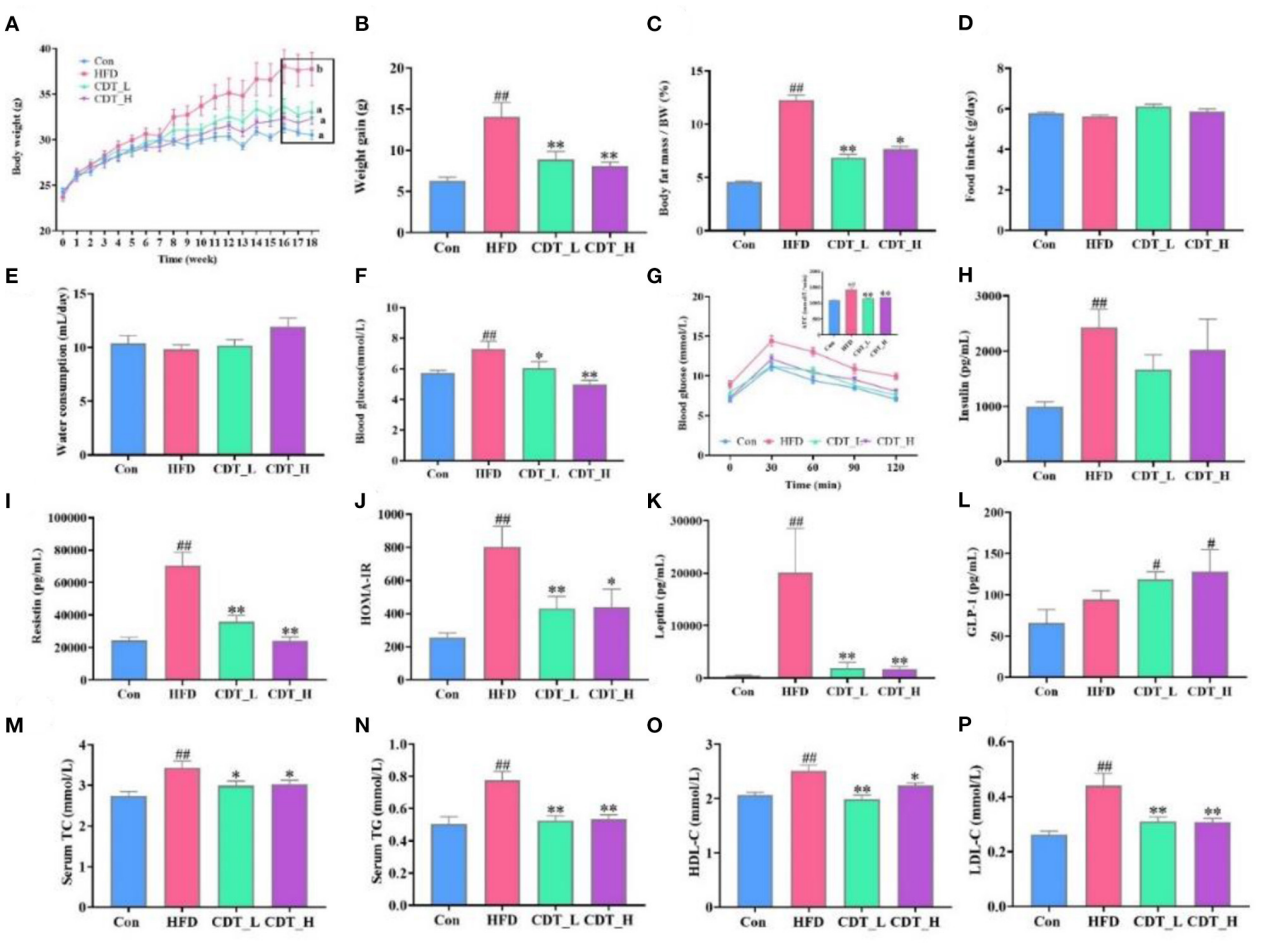
CDT improves glucose-lipid metabolism disorders in HFD-fed mice. **(A)** Body weight. **(B)** Body weight gain. **(C)** Body fat percentage. **(D)** Food intake. **(E)** Water consumption. **(F)** Blood glucose. **(G)** Oral glucose tolerance test (OGTT) curve and the area under the curve (AUC). **(H)** Insulin. **(I)** Resistin. **(J)** Homeostatic model assessment of insulin resistance (HOMA-IR). **(K)** Leptin. **(L)** Glucagon-like peptide-1 (GLP-1) **(M)** total cholesterol (TC). **(N)** Serum triglyceride (TG). **(O)** High-density lipoprotein-cholesterol (HDL-C). **(P)** Low-density lipoprotein-cholesterol (LDL-C). *N* = 9. All data are shown as mean ± SEM. *P < 0.05, **P < 0.01 by one-way ANOVA *P < 0.05, **P < 0.01 compared with that of the HFD group. ^#^*P* < 0.05, ^*##*^*P* < 0.01 compared with that of the Con group by one-way ANOVA.

C57BL/6 mice were supplemented with CDT under continuous HFD feeding for 18 weeks to investigate the anti-obesity effects of CDT in HFD-fed mice. As shown in Figures 1A, B, the CDT_L and CDT_H groups had significantly lower body weights than that of the HFD group (*P* < 0.01), which decreased by 15 and 16%, respectively. Furthermore, as expected, the body fat rate decreased by 44% in the CDT_L group, while the body fat rate in the CDT_H group decreased by 38% (Figure 1C). More notably, the difference in body weight and body fat rate was not ascribed to reduced food intake and water consumption, since no significant difference was found in food intake and water consumption between all groups (Figures 1D, E). As shown in Figure 1F, after drinking low- and high-concentrations of CDT, the fasting blood glucose levels of the CDT_L and CDT_H groups were reduced to varying degrees (*P* < 0.05 and *P* < 0.01, respectively). Similar trends were observed in the results of the OGTT among the groups; the OGTT was significantly reduced in both the CDT_L and CDT_H groups (Figure 1G, *P* < 0.01). Moreover, HFD treatment caused notable increases in the mice's insulin, resistin, HOMA-IR, and leptin values, which were restored in the CDT_L and CDT_H groups (Figures 1H–K). Compared with the Con group, drinking low- and high-concentrations of CDT significantly increased the content of GLP-1 (Figure 1L, *P* < 0.05). By measuring serum lipid markers, the serum levels of TG, TC, HDL-C, and LDL-C were higher in HFD-fed mice than in the CDT_L and CDT_H groups (Figures 1M–P).

### 3.2. Effects of CDT on fat cell morphology and lipid metabolism in HFD-fed mice

**Figure 2:**
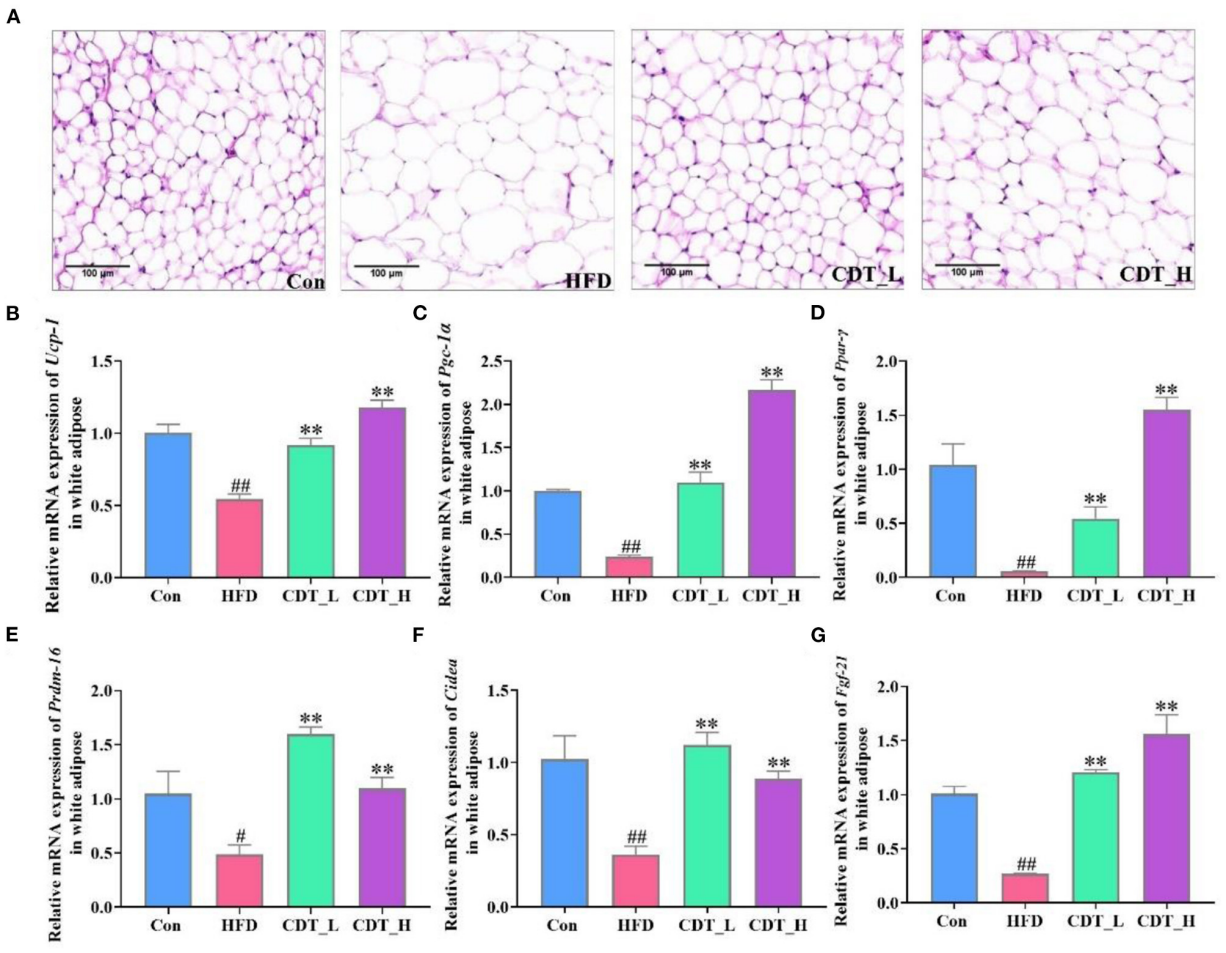
CDT improve fat cell morphology and lipid metabolism disorders of white adipose tissue in HFD-fed mice. **(A)** H&E staining of subcutaneous adipose tissue. **(B–G)** mRNA expression of *Ucp-1, Pgc-1*α, *Ppar-*γ, *Prdm-16, Cidea*, and *Fgf-21* in the white adipose tissue. *N* = 5 in A and *n* = 3 in **(B–G)**. All data are shown as mean ± SEM. **P < 0.01 compared with that of the HFD group; ^#^*P* < 0.05, ^*##*^*P* < 0.01 compared with that of the Con group by Kruskal–Wallis rank-sum test.

**Figure 3:**
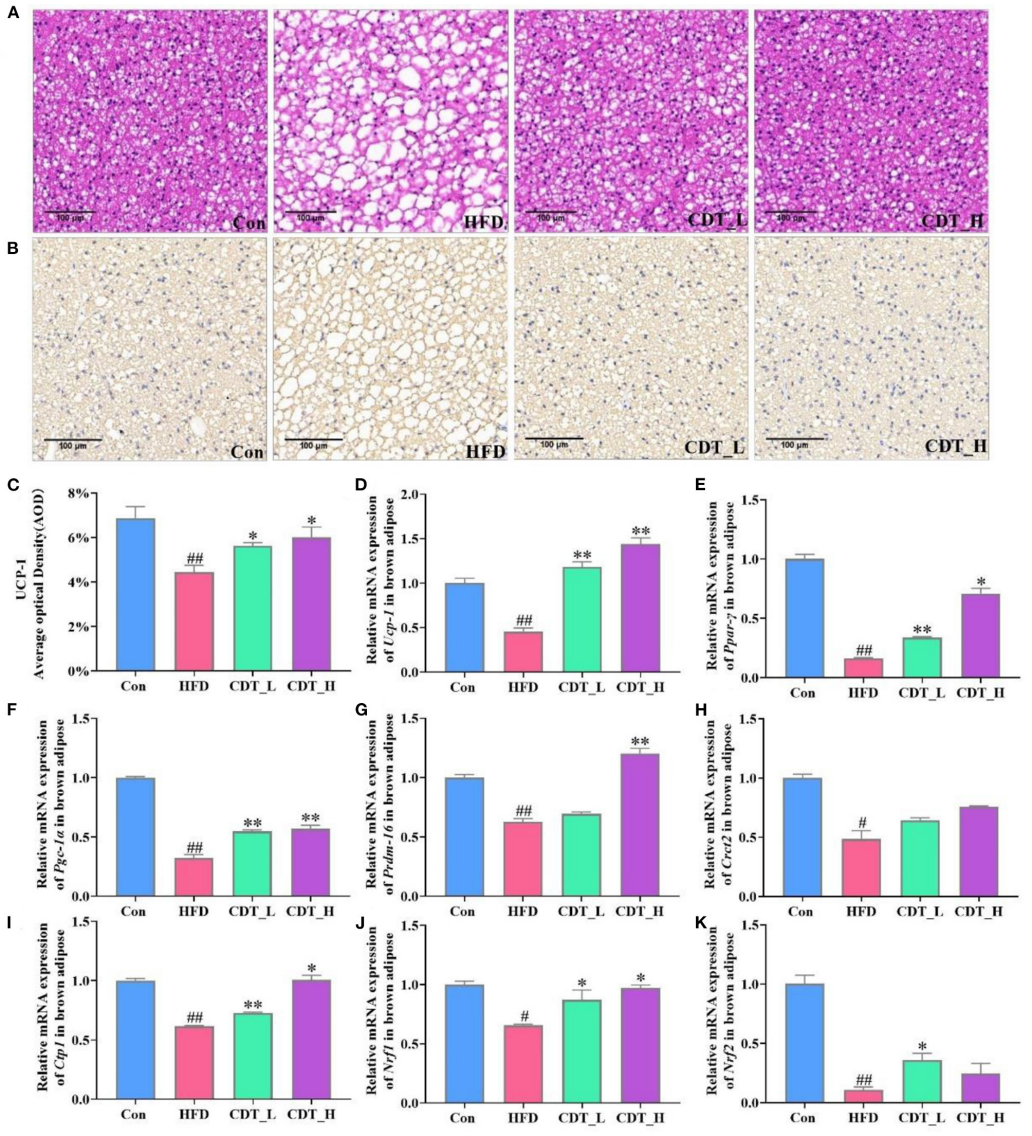
CDT improve fat cell morphology and lipid metabolism disorders of brown adipose tissue in HFD-fed mice. **(A)** H&E staining of brown adipose tissue. **(B)** Immunohistochemical analysis of Ucp-1 in the brown adipose tissue. **(C)** Average optical density of Ucp-1. D-K: mRNA expression of *Ucp-1, Ppar-*γ, *Pgc-1*α, *Prdm-16, Crct2, Ctp1, Nrf1*, and *Nrf2* in the brown adipose tissue. *N* = 5 in **(A–C)** and *n* = 3 in **(D–K)**. All data are shown as mean ± SEM. *P < 0.05, **P < 0.01 compared with that of the HFD group; ^#^*P* < 0.05, ^*##*^*P* < 0.01 compared with that of the Con group by Kruskal–Wallis rank-sum test.

When comparing obesity-related traits, we demonstrated that representative histological slices of subcutaneous adipose tissue, as presented in Figure 2A, suggested that CDT significantly lowered the mean adipocyte size in HFD-fed mice. Interestingly, the CDT_L group had a better effect on reducing the size of adipocytes than the CDT_H group. In addition, CDT was able to reduce the size of brown adipose cells on the HFD, as shown in Figure 3A, and the effect of the CDT_H group was better than that of the CDT_L group. Previous studies have reported that the body upregulates the expression of the brown adipose tissue-specific gene, uncoupling protein 1 (Ucp-1), enhances energy expenditure, and promotes browning of subcutaneous adipose tissue, preventing weight gain (35). Therefore, to determine whether the increased energy expenditure in the CDT_L and CDT_H groups was associated with adaptive thermogenesis, we assessed the effects of CDT administration on brown adipose tissue. Immunohistochemical analysis of Ucp-1 revealed that CDT administration promoted thermogenesis of brown adipose tissue compared to that in the HFD group (Figures 3B, C, *P* < 0.05). To further understand the effects of CDT intervention on lipid metabolism, we analyzed the expression of relevant genes in the white and brown adipose tissues. We observed lower mRNA expression of thermoregulatory markers and lipid metabolism-related genes, including *Ucp-1*, peroxisome proliferation-activated receptor-gamma coactivator 1 alpha (*Pgc-1*α), peroxisome proliferator-activated receptor gamma (*Ppar-*γ), PR domain containing 16 (*Prdm-16*), cell death-inducing DNA fragmentation factor alpha-like effector A (*Cidea*), and fibroblast growth factor 21 (*Fgf-21*), in the white adipose tissue of the HFD group than in the Con group. However, the CDT_L and CDT_H groups expressed higher mRNA levels of these thermoregulatory markers and lipid metabolism-related genes than the HFD group (Figures 2B–G, *P* < 0.01). Furthermore, to examine the changes in mRNA expression of thermoregulatory markers and lipid metabolism-related genes in brown adipose tissue by HFD, the mRNA expression of thermoregulatory markers and lipid metabolism-related genes was determined (Figures 3D–K). The expression of *Ucp-1, Ppar-*γ, *Pgc-1*α, *Prdm-16*, CREB-regulated transcription coactivator 2 (*Crct2*), carnitine palmitoyltransferase 1 (*Ctp1*), nuclear respiratory factor 1 (*Nrf1*), and *Nrf2* was lower in HFD-fed mice than in the Con group. However, the CDT_L group exhibited a significantly upregulated mRNA expression of *Ucp-1, Ppar-*γ, *Pgc-1*α, and *Ctp1* (*P* < 0.01) and upregulated mRNA expression of *Nrf1* and *Nrf2* compared with the HFD group (*P* < 0.05). The CDT_H group exhibited a significantly upregulated mRNA expression of *Ucp-1, Pgc-1*α, and *Prdm-16* (*P* < 0.01) and upregulated mRNA expression of *Ppar-*γ, *Ctp1*, and *Nrf1* compared with the HFD group (*P* < 0.05).

### 3.3. Effects of CDT on liver function and lipid metabolism in HFD-fed mice

**Figure 4:**
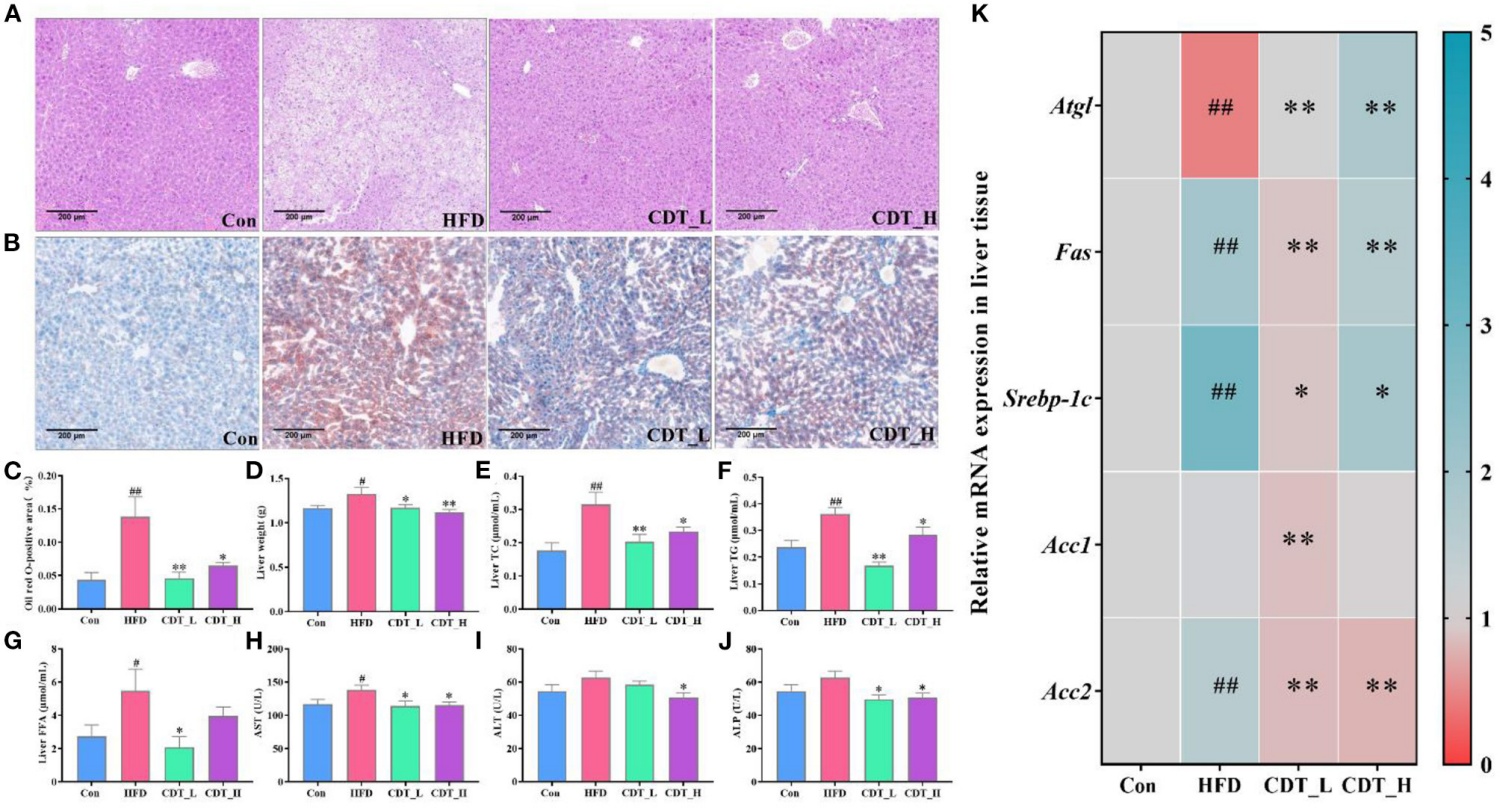
CDT improves liver function and lipid metabolism in HFD-fed mice. **(A)** H&E staining of liver tissues; **(B)** oil red O staining of liver tissues; **(C)** oil red O-positive area; **(D)** liver weight; **(E)** liver TC; **(F)** liver TG; **(G)** liver FFA; **(H)** AST activity; **(I)** ALT activity; **(J)** ALP activity; **(K)** mRNA expression of *Atgl, Fas, Srebp-1c, Acc1*, and *Acc2* in the liver. *n* = 5 in **(A–C)**, *n* = 7 in **(D–J)**, and *n* = 3 in **(K)**. All data are shown as mean ± SEM. *P < 0.05, **P < 0.01 by one-way ANOVA *P < 0.05, **P < 0.01 compared with that of the HFD group. ^#^*P* < 0.05, ^*##*^*P* < 0.01 compared with that of the Con group by one-way ANOVA (*n* = 7) and Kruskal–Wallis rank-sum test (*n* ≤ 5).

As shown in Figure 4A, H&E staining of liver sections revealed that HFD-induced indistinct cell boundaries, denaturation, irregular damaged cells with cytoplasmic vacuolation and that the CDT_L and CDT_H interventions improved this outcome. Oil red O staining revealed that CDT_L and CDT_H alleviated HFD-induced hepatic steatosis, and the effect of CDT_L was greater than that of CDT_H (Figures 4B, C). Liver weight was significantly increased in HFD-fed mice compared to that in the Con group, whereas CDT_L and CDT_H intervention remarkably lowered liver weight in mice on HFD (Figure 4D, *P* < 0.05 and *P* < 0.01, respectively). CDT_L and CDT_H also markedly reduced the levels of hepatic TC and TG in HFD-fed mice (Figures 4E, F; *P* < 0.01 and *P* < 0.05, respectively). Furthermore, CDT_L significantly reduced liver FFA levels in HFD-fed mice (Figure 4G, *P* < 0.05). The elevated levels of ALT in the HFD group were significantly ameliorated by CDT_H treatment (Figure 4I). In addition, Figures 4H, J demonstrates that CDT_L and CDT_H enhanced the increase AST and ALP levels in the HFD group, indicating that CDT_L and CDT_H exert protective effects on hepatotoxicity. Furthermore, to examine the changes in hepatic lipid metabolism regulated by HFD, the expression of lipid metabolism-related genes was determined (Figure 4K). The expression of genes, including adipose triglyceride lipase (*Atgl*), was lower in the HFD-fed mice than in the Con group. However, CDT_L and CDT_H significantly upregulated the mRNA expression of *Atgl* compared to that in the HFD group (*P* < 0.01). At the same time, the expression of sterol regulatory element-binding protein 1c (*Srebp-1c*), *Fas*, and acetyl-CoA carboxylase 2 (*Acc2*), which are related to lipid synthesis in the liver, was upregulated under HFD conditions. However, CDT_L and CDT_H significantly reduced the mRNA expression of *Srebp-1c, Fas*, and *Acc2* compared with the HFD group. In addition, the expression of Acc1 is significantly reduced in the CDT_L group compared to that in the HFD group (*P* < 0.01).

### 3.4. Effects of CDT on the gut microbiota in mice

**Figure 5:**
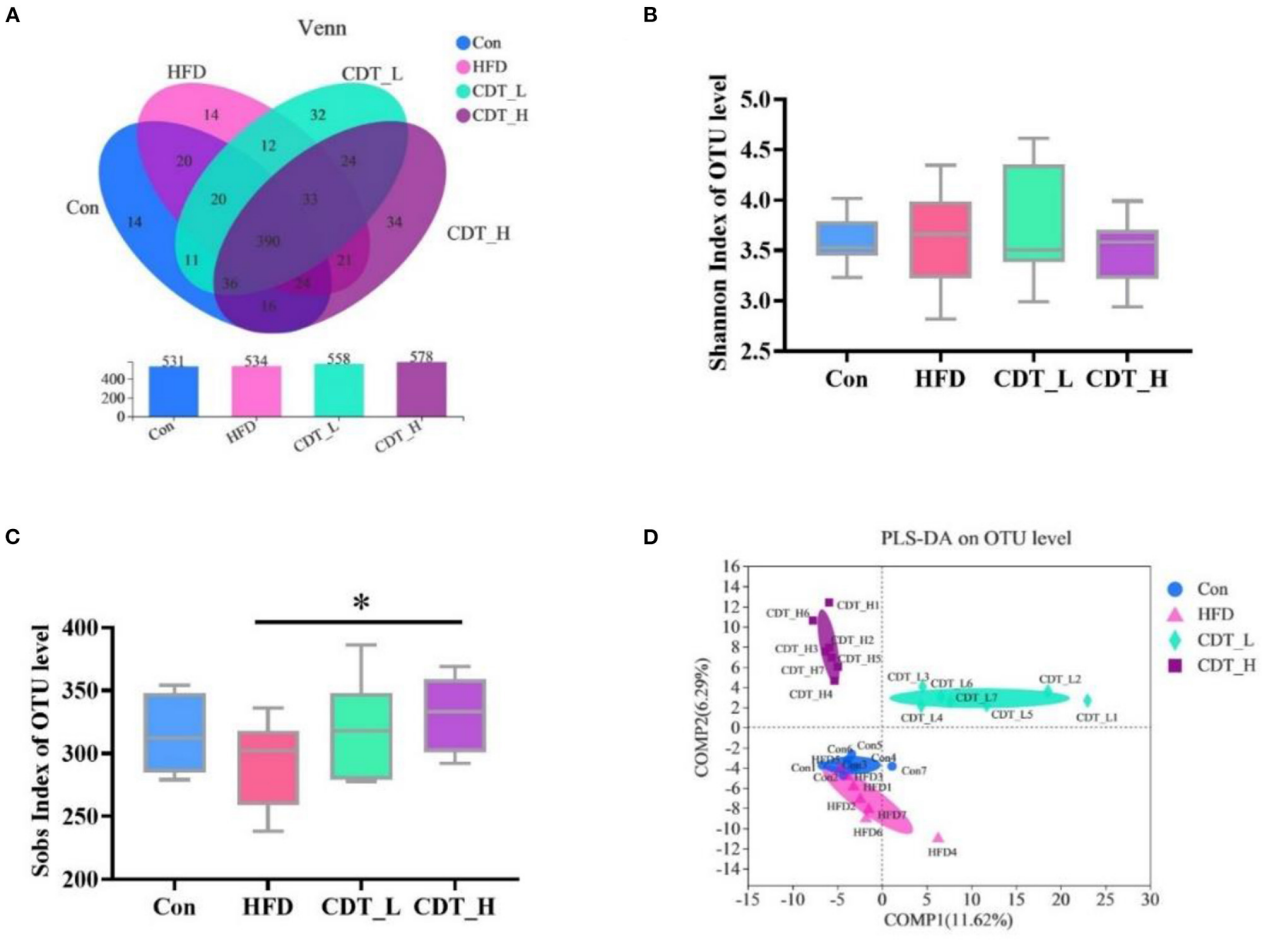
CDT alters the gut microbiota composition in HFD-fed mice. **(A)** Venn diagrams showing the unique and shared OTUs in the gut microbiota among groups. **(B)** Shannon index of OTU level. **(C)** Sobs index of OTU level. **(D)** PLS-DA on OTU level. *N* = 7. *P < 0.05 compared with that of the HFD group by one-way ANOVA.

The effects of HFD and CDT on the gut microbiota of mice were analyzed in the current study. After removing the unqualified sequence, as shown in Figure 5A, the Con group displayed 531 operational taxonomic units (OTUs), whereas the HFD, CDT_L, and CDT_H groups displayed 534, 558, and 578 OTUs, respectively. As shown in Figure 5B, the Shannon index at the OTU level was not significantly different among the four groups, but there was an upward trend in the CDT_L group compared with the other three groups. The Sobs index of OTU level results demonstrated a significant increase in the CDT_H group compared with the HFD group (*P* < 0.05). In addition, it was observed that there was a tendency to reduce the Sob index of the HFD group compared with the Con and CDT_L groups (Figure 5C). Moreover, PLS-DA analysis revealed differences between the HFD, CDT_L, and CDT_H groups (Figure 5D).

### 3.5. CDT regulates the composition of gut microbiota in mice

**Figure 6:**
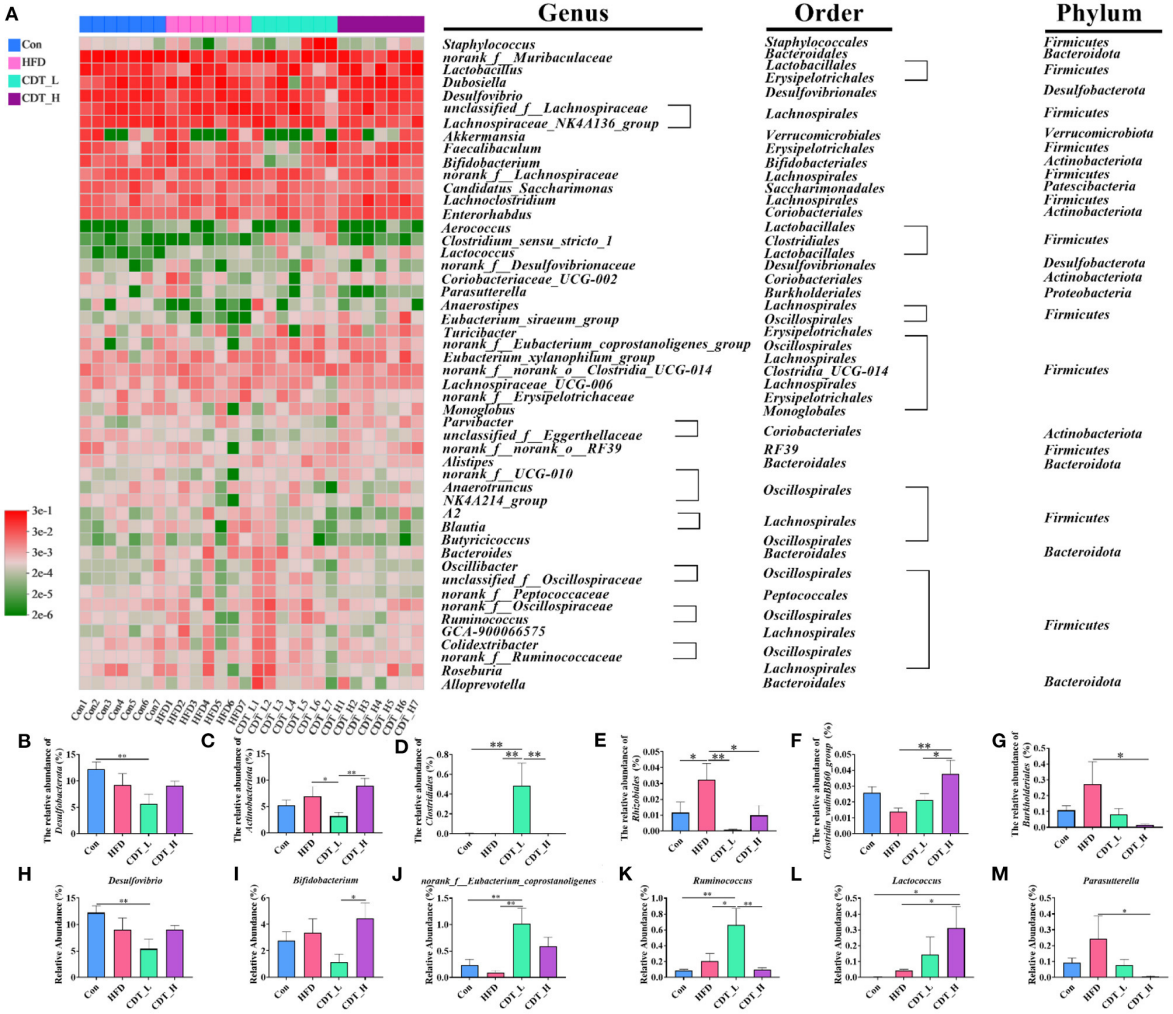
CDT regulates the gut microbiota composition in mice. **(A)** Heatmap of bacterial taxonomic profiling at the genus, order, and phylum. **(B, C)** Relative abundance of cecal *Desulfobacterota* and *Actinobacteriota* at the phylum level in each group. **(D–G)** Relative abundance of *Clostridiales, Rhizobiales, Clostridia_vadinBB60_group*, and *Burkholderiales* at the order level in each group. **(H–M)** relative abundance of six representative genera. *N* = 7. *P < 0.05, **P < 0.01 by one-way ANOVA.

To explore differences in gut microbial composition, the relative abundance of bacterial communities at the phylum and genus levels was analyzed. At the phylum level, the gut flora of the four groups was dominated by *Firmicutes, Bacteroidota, Desulfobacterota*, and *Actinobacteria* (Figure 6A). As shown in Figure 6B, at the phylum level, *Desulfobacterota* accounted for 12.25% of the intestinal flora in the Con group, and this type of bacteria constituted 5.64% of the microbial profile in the CDT_L group. Actinobacteria accounted for 8.95% of the intestinal flora in the CDT_H group and 3.17% of the microbial profile in the CDT_L group (Figure 6C). At the order level, an increased abundance of *Clostridiales* was observed in the CDT_L group (Figure 6D). In addition, high dietary fiber intake significantly increased the relative abundance of *Rhizobiales* in HFD-fed mice (Figure 6E). High concentrations of CDT significantly increased the relative abundance of *Clostridia_vadinBB60_group*, compared with those in both the HFD and CDT_L groups (Figure 6F). Compared with the HFD group, the relative abundance of *Burkholderiales* in the CDT_H group decreased (*P* < 0.05) (Figure 6G). At the genus level, the Con group possessed significantly higher levels of *Desulfovibrio* than did the CDT_L group (Figure 6H). Meanwhile, high concentrations of CDT led to an increase in the relative abundance of *Bifidobacterium* compared to the CDT_L group (Figure 6I). In addition, the low concentration of CDT markedly increased the relative abundances of multiple genera, including *norank_f__Eubacterium_coprostanoligenes* and *Ruminococcus*, when compared to the HFD group (Figures 6J, K). The CDT_H group possessed significantly higher levels of *Lactococcus* but lower levels of *Parasutterella* than those in the HFD group (Figures 6L, M).

### 3.6. Correlation analysis of intestinal microbiota with phenotype in mice

**Figure 7:**
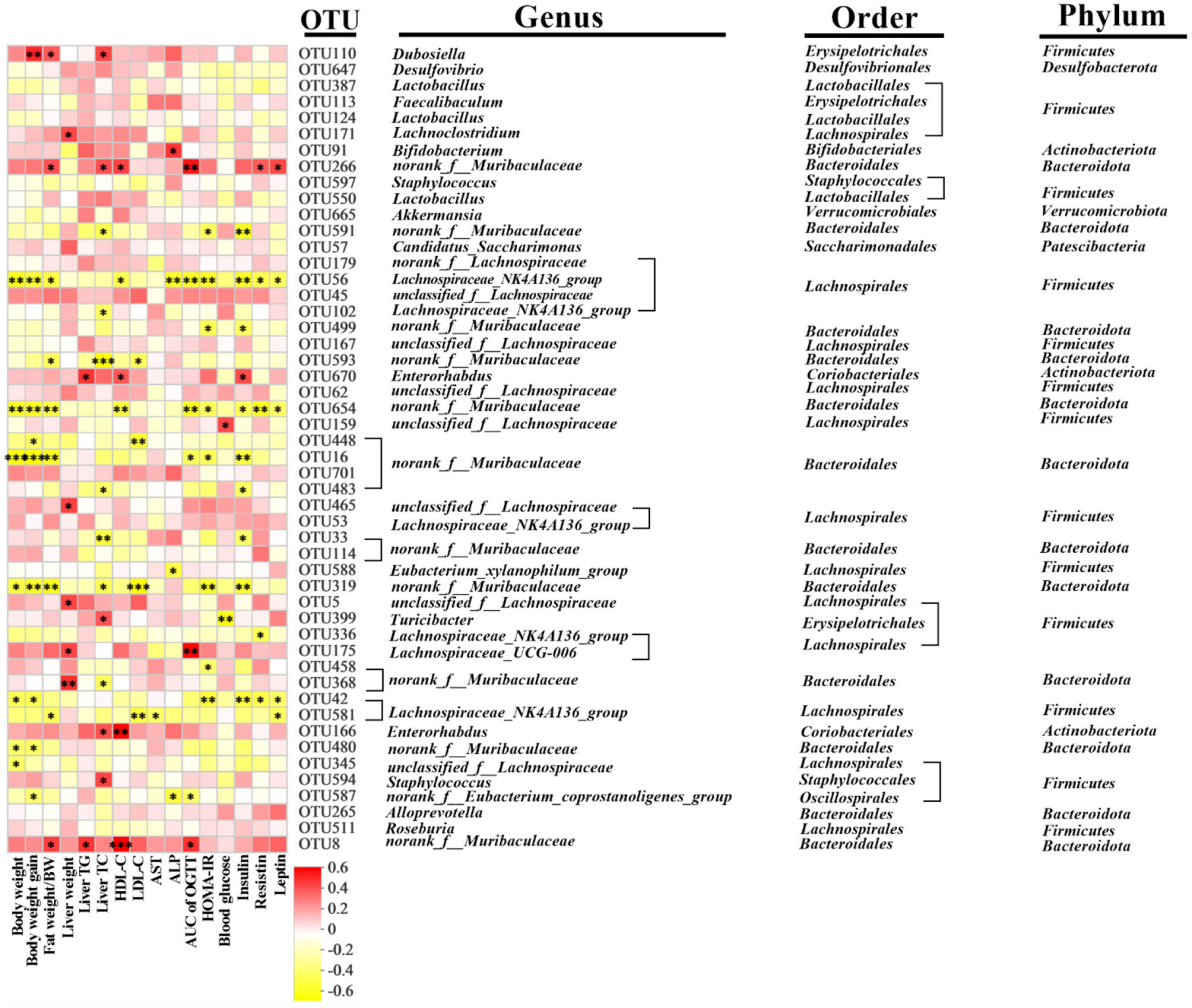
Predictive analysis of intestinal microbiota phenotype in mice. Heatmap of Spearman's correlation between OTUs and indexes related to obesity-related indexes in mice altered by HFD or CDT intervention. A total 33 OTUs were significantly correlated at least with one of the parameters including body weight, fat percentage, weight gain, AUC of OGTT, liver weight, liver TC, liver TG, HDL-C, LDL-C, AST, ALP, HOMA-IR, blood glucose, insulin, resistin, and leptin. *N* = 7. *P < 0.05, **P < 0.01 by one-way ANOVA.

Spearman's correlation analysis was performed to understand the association between differentially enriched microbes and obesity-related traits (Figure 7). Nineteen and 12 OTUs were negatively and positively correlated with obesity-related traits, respectively. *Bifidobacterium* (OUT 91) was positively correlated with ALP. Low-concentration CDT significantly increased the relative abundance of *Bifidobacterium* compared to that in the CDT_H group (Figure 6I). *norank_f__Eubacterium_coprostanoligenes* (OUT 587) was observed to be negatively correlated with the weight gain, ALP, and AUC of the OGTT. Meanwhile, low concentrations of CDT led to an increase in the relative abundance of *norank_f__Eubacterium_coprostanoligenes* compared to the Con and HFD groups (Figure 6J). The gut flora of the two groups were dominated by *norank_f__Muribaculaceae* and *Lachnospiraceae_NK4A136_group* at the genus level. The *norank_f__Muribaculaceae* was negatively correlated with body weight, fat percentage, weight gain, AUC of OGTT, liver TC, HDL-C, LDL-C, HOMA-IR, insulin, resistin, and leptin, including OUTs 591, 499, and 593, OTU 654, and OUTs 448, 16, 483, 33, 319, 458, and 480. Similarly, the *Lachnospiraceae_NK4A136_group* was negatively correlated with body weight, fat percentage, weight gain, AUC of OGTT, liver TC, HDL-C, LDL-C, AST, ALP, HOMA-IR, blood glucose, insulin, resistin, and leptin, including OUTs 56, 102, 336, 42, and 581. Taken together, these results suggest that the low-concentration CDT intervention modulates HFD-induced gut microbiota dysbiosis in mice.

## 4. Discussion

It is well-known that dark tea contains polysaccharides, polyphenols, and alkaloids, which have various effects on the body, including anti-inflammatory, antioxidant, lipid-lowering, and antiobesity effects (36). In our experiments, CDT was based on Fu brick tea, to which green tea, *Ilex latifolia Thunb* (kuding tea), and *Momordica grosvenori* (Luo Han Guo) were added to improve the taste and antiobesity effects of CDT. Consistent with previous studies, mice fed a HFD for 18 weeks demonstrated that CDT had lipid-lowering and antiobesity effects (37). It is noteworthy that, in this study, low-concentration CDT prevented abdominal obesity in mice better than high-concentration CDT, setting the stage for future decisions on the concentration of CDT consumption. Yang and Hong (38) also demonstrated that drinking tea was a good resistance to abdominal obesity, based on the lack of a significant difference in feed intake.

Obesity usually leads to an increase in the level of blood glucose and insulin resistance, which are regarded as early symptoms of diabetes (39). Meanwhile, blood glucose levels are key indicators of diabetes (40). CDT was demonstrated to positively regulate blood glucose levels in HFD-fed mice by measuring fasting blood glucose levels and performing the OGTT. The results of diabetes-related indicators demonstrate that, although HFD can increase the content of insulin, the significant increase in the resistin and insulin resistance index indicated that HFD led to a significant decrease in the sensitivity of mice to insulin and that long-term drinking of CDT can reduce the resistin and insulin resistance index and improve insulin sensitivity. In a study of sweet tea, it was also demonstrated that sweet tea leaf extract reduced the resistin and leptin levels (41). Leptin is a product of obesity, plays an important role in regulating food intake, body weight, and lipolysis (42), and is positively correlated with leptin content (43). In our study, leptin levels in HFD-fed mice increased significantly, and drinking the two concentrations of CDT led to a significant decrease in leptin levels. GLP-1 is secreted after eating, reducing blood glucose concentration by increasing insulin secretion and inhibiting glucagon release (44). Drinking CDT can significantly increase the GLP-1 content, which is similar to the results for resistin and leptin, demonstrating that CDT can increase insulin sensitivity and decrease blood glucose. In addition, consistent with previous studies, serum levels of TC, TG, LDL-C, and HDL-C were reduced in the CDT group (45). These results demonstrate that CDT has lipid-lowering and antiobesity effects in mice; however, the underlying mechanism requires further study.

The primary locations for lipid metabolism are adipose tissue and the liver, where energy metabolism, lipogenesis, and obesity are regulated (46). Obesity is caused by excessive accumulation of fat and is often accompanied by disorders of glucose and lipid metabolism (47). In our study, CDT decreased the area of subcutaneous fat and brown fat cells induced by the HFD. Immunohistochemical analysis revealed that CDT increased brown fat Ucp-1. Similar to our results, it was demonstrated that administering white tea to rats with removed ovaries activated mRNA expression of brown adipose tissue Ucp-1 in a trial of white tea (48). Brown fat is a special adipose tissue rich in mitochondria, and its activity is inversely correlated with obesity, blood glucose concentration, and insulin sensitivity (49). Ucp-1 plays a vital role in thermogenesis and energy regulation in brown fat (50). Brown fat, a good fat, is usually obtained by browning white fat through different pathways. *Ppar-*γ is the primary transcriptional regulator of fat differentiation and activates *Pgc-1*α with *Prdm-16* to induce brown fat gene expression (51–53). Additionally, fat browning is driven by *Fgf-21* (54). As a result, white fat is converted to beige fat, which eventually becomes brown fat. Beige fat has higher levels of *Ucp-1* and *Cidea* than white fat (55, 56). In the present study, CDT upregulated the expression of key transcription factors (*Ucp-1, Pgc-1*α, *Ppar-*γ, *Prdm-16, Cidea*, and *Fgf-21*), thereby promoting the browning of white fat. In addition, the activation of *Nrf2* increases energy consumption and facilitates the browning of white fat to prevent obesity (57). *Nrf1*, a key gene for maintaining brown fat, is the basic regulator of brown adipose tissue that increases metabolism in cold or obese conditions through proteasome function (58). *Nrf1* and *Nrf2* were significantly up-regulated in the CDT_L group. In addition, a significant upregulation of *Ctp1* can increase energy consumption (59), and *Ctp1* is significantly upregulated following the CDT intervention. These results demonstrate that CDT can accelerate energy consumption and the browning of white fat to reduce fat and ameriolate obesity. The liver is the central organ for fatty acid metabolism, and under normal circumstances, it only has a small amount of fat. Excess nutrition or obesity leads to liver function damage, lipid metabolism disorders, and accumulation of triglycerides in the liver cells (60). AST, ALT, and ALP levels in the HFD group increased in this trial, indicating that the HFD caused liver function impairment (61). To further verify whether impairment of liver function causes lipid deposition, we examined the levels of liver TC, TG, and FFA. The results demonstrated that the TC, TG, and FFA levels were significantly increased, indicating that HFD led to a large amount of lipid deposition in the liver, which is consistent with the results of previous studies (62). The two concentrations of CDT improved lipid deposition and liver function, and the effect of low-concentration CDT was better than that of high-concentration CDT. Tea and its extracts have also been demonstrated to exert protective effects on liver function and lipid metabolism (63). The inhibition of hepatic steatosis is associated with many mechanisms, including reduced fat production, increased fatty acid β-oxidation, increased insulin sensitivity, the inhibition of oxidative stress, and the inhibition of activation of the inflammatory pathway (64). To explore this mechanism, the molecular mechanisms underlying liver lipid metabolism were investigated. Activation of AMP-activated protein kinase (AMPK) plays a significant role in regulating lipid formation and fatty acid oxidation in adipose tissue (65), and lipogenesis in the liver is mainly regulated by *Srebp-1c*, which further regulates the expression of *Acc1*, the main regulator of lipid biosynthesis by inhibiting *Srebp-1c* in the nucleus (66). Both high- and low-concentration CDT inhibited the expression of *Srebp-1c* compared with the HFD, but only low-concentration CDT significantly inhibited the expression of *Acc1*. Fat synthesis also relies on the activation of *Acc2* (67), and both high- and low-concentration CDT can significantly reduce the mRNA expression of *Acc2*. In addition, *Fas* and *Srebp-1c* have the same effect on fat synthesis (68), and low concentrations of CDT can significantly reduce the relative expression of *Fas*, indicating that low concentrations of CDT significantly reduce hepatic lipogenesis. In addition to the discovery of the inhibitory effect of CDT on hepatic lipogenesis, we also demonstrated that high and low doses of CDT could activate the expression of *Atgl* genes (69), which are related to lipolysis, accelerating the metabolism of liver fat. Combining the above results, it can be concluded that CDT can increase liver fat metabolism and inhibit liver fat synthesis, thereby improving liver lipid deposition and lipid metabolism disorders induced by HFD.

An increasing amount of research demonstrates that the intestinal flora plays an important role in human health (70). In general, experimental studies on obesity and intestinal flora include an HFD model group, and the intestinal flora of the HFD model group will change (15). In our experiment, we demonstrated that HFD decreased the species richness of the gut microbiota compared with CDT_H (71). In addition, PLS-DA analysis revealed that the HFD group and the two concentrations of CDT had different intestinal flora. Interestingly, mice in the Con and HFD groups had the most similar intestinal profiles. We suspect that the PLS-DA analysis results may be because most of the microbiota in the Con and HFD groups did not differ significantly at the phylum and genus levels and that most of the differences were generated between the drinking compound dark tea and the drinking water group. By analyzing the differences in the flora at the genus level in our study, it was demonstrated that a low concentration of CDT was able to reduce the level of *Desulfovibrio*, which has been demonstrated to be harmful in previous studies, and the endotoxin of *Desulfovibrio* was associated with inflammation; therefore, *Desulfovibrio* was positively associated with inflammation and obesity (72). Few studies have examined the function of the *norank_f__Eubacterium_coprostanoligenes genus*, but it has been observed that adding perilla fruit leaves to dairy cow feed can increase the abundance of *Eubacterium coprostanoligenes*, and the abundance of this bacterium is inversely correlated with dairy cow fat (73), which is similar to the results of the CDT_L group in our study. In addition, the abundance of the genus *Ruminococcus* in the CDT_L group was higher than that in the other three groups, and multiple studies have demonstrated that the abundance of *Ruminococcus* is inversely correlated with obesity (74, 75). As a beneficial bacterium, *Bifidobacterium* can alleviate obesity, regulate glucose homeostasis, and produce short-chain fatty acids (SCFAs) (76, 77). In addition, a previous study has demonstrated that the growth of *Bifidobacterium* can be promoted by diet (78). In our study, the high concentration of CDT increased the abundance of *Bifidobacterium*. In addition, the abundance of *Lactococcus* in the CDT_H group was higher than that in the Con and HFD groups, which was consistent with a previous study demonstrating that the abundance of *Lactococcus* is inversely correlated with obesity (79). In addition, the abundance of *Parasutterella*, which is associated with chronic intestinal inflammation, was decreased in the CDT_H group (80).

## 5. Conclusion

**Figure 8:**
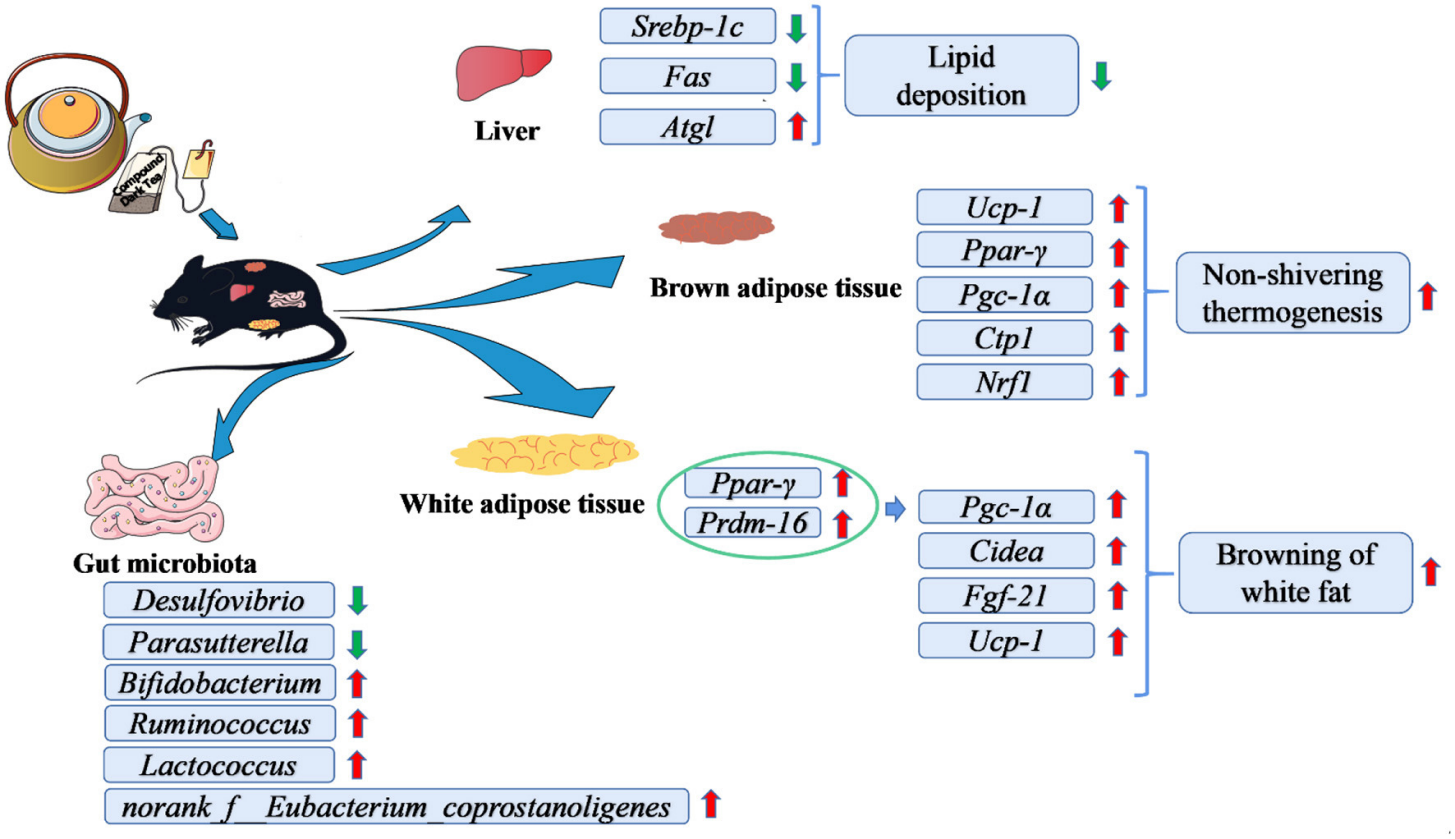
Schematic diagram of the effects of CDT in alleviating high-diet-induced obesity and hepatic steatosis and modulating the gut microbiota.

The schematic diagram of CDT is concluded in Figure 8. In this study, we found that CDT upregulated the expression of thermogenic genes in brown fat and browning-related genes in white fat tissues. CDT also significantly upregulated the expression of lipolysis-related genes and downregulated that of lipid synthesis-related genes in the liver. As a result, the CDT reduced fat deposition and alleviated hepatic steatosis in HFD-fed mice. Additionally, CDT increased the abundance of beneficial bacteria and reduced the abundance of harmful bacteria in the intestine. This study suggests that CDT can significantly ameliorate lipid metabolism in mice and opens new avenues for the development of functional compound tea drinks.

## Data availability statement

The datasets presented in this study can be found in online repositories. The names of the repository/repositories and accession number(s) can be found in the article/ Supplementary material.

## Ethics statement

The animal study was reviewed and approved by the Ethics Committee of Hunan Agricultural University.

## Author contributions

JQ and MY designed the experiment. CW is the first inventor of the compound dark tea patent. JQ wrote original draft. MY, JQ, and CW prepared the experiment and made all H&E and oil red staining sections. JQ, XL, LZ, and ML analyzed the data and prepared for graph. JW and LW wrote original draft and reviewed manuscript. JW reviewed manuscript and checked the grammar. LW and ZL reviewed manuscript and provided funding acquisition. All authors have read and agreed to the published version of the manuscript.
