# Supplementary material for: Compound dark tea ameliorates obesity and hepatic steatosis and modulates the gut microbiota in mice
Source: Front Nutr. 2023 Jan 20;10:1082250. doi: 10.3389/fnut.2023.1082250 (PMC9895393; doi:10.3389/fnut.2023.1082250)
Supplement: Supplementary file 1 [file Table_1.DOCX]

**Table S1. Diet feed composition**

| Ingredients | Content（%） | |
| --- | --- | --- |
|  | 15% level | 35% level |
| basic feed | 98.4 | 77.2 |
| protein | 0.6 | 8.5 |
| sucrose | 0.1 | 0.1 |
| Oil | 0.8 | 11.8 |
| cellulose | 0 | 0.6 |
| Cellulose, mineral | 0.1 | 1.7 |
| total | 100 | 100 |

**Table S2. Dietary feed calorie level**

| heat density（kcal/g） | 3.5 | 4 |
| --- | --- | --- |
| protein | 22.0% | 22.0% |
| carbohydrate | 63.0% | 43.0% |
| lipid | 15.0% | 35.0% |
| total | 100.0% | 100.0% |

**Table S3. Main reagents and antibodies**

| [**Reagent**](javascript:;)**s/ Antibodies** | [**Manufacturers**](javascript:;) | **Product number** | **Addresses** |
| --- | --- | --- | --- |
| Triglyceride (TG) assay kit | Mindray Bio-Medical Electronics |  | Shenzhen, China |
| Cholesterol (TC) assay kit | Mindray Bio-Medical Electronics |  | Shenzhen, China |
| High-density lipoprotein cholesterol (HDL-C) assay kit | Mindray Bio-Medical Electronics |  | Shenzhen, China |
| Low-density lipoprotein cholesterol (LDL-C) assay kit | Mindray Bio-Medical Electronics |  | Shenzhen, China |
| Alkaline phosphatase (ALP) assay kit | Mindray Bio-Medical Electronics |  | Shenzhen, China |
| Aspartate transaminase (AST) assay kit | Mindray Bio-Medical Electronics |  | Shenzhen, China |
| Blood glucose test strips | ACCU- CHEK, Roche |  | Shanghai, China |
| Insulin (Mouse Diabetes 8-Plex Assay) | Bio-Rad Laboratories | 171F7001M | Shanghai, China |
| Resistin (Mouse Diabetes 8-Plex Assay) | Bio-Rad Laboratories | 171F7001M | Shanghai, China |
| Leptin (Mouse Diabetes 8-Plex Assay) | Bio-Rad Laboratories | 171F7001M | Shanghai, China |
| GLP-1 (Mouse Diabetes 8-Plex Assay) | Bio-Rad Laboratories | 171F7001M | Shanghai, China |
| Triglyceride (TG) content assay kit | Beijing Box Shenggong Technology | AKFA003M | Beijing, China |
| Free fatty acid (FFA) content assay kit | Beijing Box Shenggong Technology | AKFA008C | Beijing, China |
| [Total cholestenone(TC) content assay kit](http://www.bioon.com.cn/reagent/show_product.asp?id=7874060) | Beijing Solarbio Science & Technology | BC1980 | Beijing, China |
| Hematoxylin-eosin (H&E) staining solution | Wuhan Servicebio | G1005 | Wuhan, Hubei, China |
| Developing and fixing reagent | Wuhan Servicebio | G2019 | Wuhan, Hubei, China |
| The prime script RT reagent kit | Takara | RR047Q | Shiga, Japan |
| SYBR Green I fluorescent dyes | Takara | RR420A | Shiga, Japan |
| Horseradish peroxidase (HRP)-conjugated secondary antibodies | AiFang biological | AFIHC001 | Changsha, Hunan, China |

**Table S4 Primer sequences used for RT-qPCR**

| Gene | Primer sequences (5’-3’) |
| --- | --- |
| *β-actin* | F: CATCCGTAAAGACCTCTATGCCAAC  R: ATGGAGCCACCGATCCACA |
| *Pgc-1α* | F: GTGTGTGCTGTGTGTCAGAG  R: AACCAGAGCAGCACACTCTAT |
| *Nrf1* | F: CTGCAGGTCCTGTGGGAAT  R: GGCTCTGAGTTTCCGAAGCA |
| *Nrf2* | F: AGGCCGTGTTAAGGAATCTGC  R: TATCCAGGGCAAGCGACTCA |
| *Ucp-1* | F: CACGGGGACCTACAATGCTT  R: ACAGTAAATGGCAGGGGACG |
| *Ppar-γ* | F: TTCGGAATCAGCTCTGTGGA  R: CCATTGGGTCAGCTCTTGTG |
| *Atgl* | F: GGGTGCGCTATGTGGATGG  R: CTCTCGCCTGAGAATGGGG |
| *Srebp-1c* | F: GATGTGCGAACTGGACACAG  R: CATAGGGGGCGTCAAACAG |
| *FAS* | F: AGGTGGTGATAGCCGGTATGT  R: TGGGTAATCCATAGAGCCCAG |
| *Acc1* | F: ATGGGCGGAATGGTCTCTTTC  R: TGGGGACCTTGTCTTCATCAT |
| *Acc2* | F: AGGTTCCAGATGCTAATGGGT  R: GCCCAGGATAAAGCTGGTCAT |
| *Crct2* | F: ACAGCCTCAGCGTCCAATC  R: GTGGAGCCGATGTCCATCAT |
| *Fgf-21* | F: GTGTCAAAGCCTCTAGGTTTCTT  R: GGTACACATTGTAACCGTCCTC |
| *Prdm-16* | F: CATGTGCGAAGGTGTCCAAA  R: GTCACCGTCACTTTTGGCT |
| *CTP1* | F: TTGGGCCGGTTGCTGAT  R: GTCTCAGGGCTAGAGAACTT |
| *Cidea* | F: TGACATTCATGGGATTGCAGAC  R: CATGGTTTGAAACTCGAAAAGGG |
